# Friction on a single MoS_2 _nanotube

**DOI:** 10.1186/1556-276X-7-208

**Published:** 2012-04-10

**Authors:** Janez Jelenc, Maja Remskar

**Affiliations:** 1 Jozef Stefan Institute, Jamova 39, Ljubljana SI-1000, Slovenia; 2 Center of Excellence NAMASTE, Jamova 39, Ljubljana SI-1000, Slovenia

**Keywords:** MoS_2_, Nanotubes, Atomic force microscopy, Low-friction nanomaterials, Tribology, Rolling process, Nanoparticles

## Abstract

Friction was measured on a single molybdenum disulfide (MoS_2_) nanotube and on a single MoS_2 _nano-onion for the first time. We used atomic force microscopy (AFM) operating in ultra-high vacuum at room temperature. The average coefficient of friction between the AFM tip and MoS_2 _nanotubes was found considerably below the corresponding values obtained from an air-cleaved MoS_2 _single crystal or graphite. We revealed a nontrivial dependency of friction on interaction strength between the nanotube and the underlying substrate. Friction on detached or weakly supported nanotubes by the substrate was several times smaller (0.023 ± 0.005) than that on well-supported nanotubes (0.08 ± 0.02). We propose an explanation of a quarter of a century old phenomena of higher friction found for intracrystalline (0.06) than for intercrystalline slip (0.025) in MoS_2_. Friction test on a single MoS_2 _nano-onion revealed a combined gliding-rolling process.

PACS, 62.20, 61.46.Fg, 68.37 Ps

## Background

Inorganic solid lubricant molybdenum disulfide (MoS_2_) is a known lubricant, which has been applied extensively for decades. Its crystalline microstructures, tribological properties, and anti-corrosive mechanisms have been studied deeply [1]. The easy mutual gliding of MoS_2 _layers along (001) basal planes and its surface inertness allow its low friction performance. Ultra-low friction coefficients as low as 0.003 between MoS_2 _flakes and MoS_2 _surfaces have been reported [2] and explained by an easy shear of basal planes of the crystal structure parallel to the sliding direction in accordance with the Amontons-Coulomb law.

MoS_2 _exists in two very different morphologies, both with a special effect on the tribology process. The usual platelike form, which can be synthesized or exploited as mineral, is widely used as an efficient dry lubricant or an oil or grease additive. Unfortunately, edges of layered crystals with high hardness are prone to oxidation which reduces the efficiency of lubrication, especially in humid environment. Thin flakes with a high active surface and with relatively low number of unsaturated bonds at edges are therefore preferable. The lubrication mechanism of MoS_2 _nanosheets, 50-nm thick, prepared by exfoliation and restacking, and added to liquid paraffin, was explained by the higher surface energy of MoS_2 _nanosheets, with better absorbance on the rubbing surfaces preventing them from a direct contact [3].

Curved, self-terminated shapes as nanotubes and fullerene-like particles (IF) with nano-onion morphology, firstly reported in 1992 on WS_2 _[4] and one year later on MoS_2 _[5], brought 'elimination' of edges and immediately became intensively investigated with regard to their particular appropriateness for a new generation of lubricants. After the first enthusiastic suggestion of a possible rolling mechanism [6,7], it was evidenced that under mechanical stress, the nanoparticles slowly deform and exfoliate, transferring WS_2 _nanosheets onto the underlying surfaces (third body effect), and continue to provide effective lubrication until they are totally exfoliated [8,9]. Another positive effect of this new nano-lubricant is that the metal surface impregnated with IF nanoparticles does not seem to oxidize during the tribological test, although the coverage of the metal surface by the nanoparticles does not exceed 20% to 30%. This observation was explained by a lower temperature of the WS_2_-impregnated interface relating to the pure metal surface during the friction test. It was furthermore suggested that the WS_2 _nanoparticles may act as a kind of 'cathodic protection' against the oxidation of the metal surface, which prevents the oxidation of the metal surface [10].

Thin films of hollow MoS_2 _nano-onions, deposited by a localized high-pressure arc-discharge method, exhibited an ultra-low friction (an order of magnitude lower than for sputtered MoS_2 _thin films) and wear in nitrogen and at 45% humidity. The results were explained by the presence of curved S ± Mo ± S planes that prevent oxidation (absence of edges) and preserve the layered structure [9]. Similarly, the experiments under boundary lubrication demonstrated that IF-MoS_2 _nanoparticles had significantly lower friction than the MoS_2 _films prepared by pulsed laser deposition [11]. The effect was explained through orientation relationship of low friction (001) MoS_2 _planes with regard to the counter face and by chemical inertness of self-terminated layers.

At deviation from fully parallel orientation between the MoS_2 _basal plane, the cleavage takes place [12] with coefficient of friction (COF) of the order of 0.1; when intracrystalline shear between MoS_2 _layers took place, the COF was found unexpectedly higher (0.06) than when intercrystalline slip occurred (0.025).

In this work, we report on the first tribo-testing performed on a single MoS_2 _nanotube and on a single MoS_2 _nano-onion using atomic force microscopy (AFM) in ultra-high vacuum (UHV) at room temperature. The MoS_2 _nanotubes with incorporated MoS_2 _nano-onions were selected because no friction experiments are done yet on such morphology, which, besides unique inorganic peapod morphology, also answers health concerns regarding release of nanoparticles into the atmosphere. Nano-onions are safely stored inside long nanotubes in non-agglomerated stage and cannot become airborne. This particular morphology of MoS_2 _nanotubes named 'mama'-tubes is made clear using transmission electron microscopy and scanning tunneling microscopy (STM) with a special attention on surface and near surface structure. Dependency of friction on load was measured at different scan areas and angles with regard to the tube axis. Nanotubes with various adhesion supports to the substrate were tested with the aim to explain a large standard deviation in friction results using nanotubes as lubricants. Results explaining a quarter of a century old phenomena of higher friction was found between MoS_2 _counterparts when shear was taking place for intracrystalline slip than for intercrystalline slip. The results obtained on single MoS_2 _nanotubes are correlated with those obtained at the same conditions on MoS_2 _single crystal and on graphite. We present new evidence that a rolling mechanism of MoS_2 _fullerene-like nano-onions is possible at low loads.

## Methods

MoS_2 _nanotubes for nanotribology testing were dispersed in ethanol using ultrasound bath and were drop casted onto freshly cleaved highly ordered pyrolytic graphite (HOPG) surface. The substrate consisted of atomically flat terraces separated by cleavage steps of different, uncontrolled height. The sample was transferred into a UHV chamber (base pressure in a range of 10^-10 ^mbar) equipped with a commercial AFM/STM (VT-AFM, Omicron Nanotechnology GmbH, Taunusstein, Germany). UHV-AFM investigations were carried out at room temperature. Single crystal silicon tip (NT-MDT, Moscow, Russia), CSG10, Sb-doped, n-type, force constant = 0.1 N/m, and curvature radius typically 10 nm, was used as AFM probe. The friction measurements were made on a top of every single nanotube. The AFM was operated in contact mode, and hence, the cantilever deflections included both the topographic and lateral force information from sample surfaces. The topographic images were used to measure the size of MoS_2 _mama-tubes, while recording the lateral deflection of the cantilever during the scanning of the 'friction images' was obtained. Measurement of the frictional force was done for at least three step increments of normal load at a given location. Lateral force image was averaged over the scanned area for both trace and retrace scans. The friction versus normal force was fitted with a straight line whose slope is the friction coefficient. Accuracy of a particular measurement was obtained from least-square fit, while mean values are shown with standard deviations.

## Results and discussion

### Results

The counterparts in the experiments consisted of the AFM tip and the top surface of nanotubes. The Hertzian contact improves parallel orientations between surface and subsurface MoS_2 _layers of thin-walled nanotubes and prevents cleavage. The contact pressure and area are dependent on the sharpness of the tip. However, when the tip scans the overside surface of a tube, cleavage of surface layers is expected, and transfer of the layered materials onto the tip could take place. The tip shape was therefore regularly reconstructed using the Scanning Probe Image Processor (SPIP) before and after each friction test. While pristine AFM tip before the friction test revealed an 8 ± 1-nm curvature radius in *x*-direction, after the test at the 3-nN load, the calculated radius was 17 ± 1 nm and increased up to 25 nm after several friction tests. Although one could expect low values of COF due to tip contamination, the value of COF over 14 measurements on the same tube, 330 nm in diameter using the same tip, was found to be 0.07 ± 0.01. The transferred material enlarged the tip wideness, but due to easy gliding, it was pushed out of the contact area, leaving the top tip intact.

Friction was measured either on areas much smaller than the diameter of the tubes, which was determined from height profiles or on areas larger than the tube diameters, but smaller than the curvature radius of the AFM tip. No convincing influence on friction by a direction of scanning with regard to a tube axis was found. The COF (*μ*) was determined using the Coulomb law, which says that friction force (friction) is proportional to the applied load, i.e., the normal force (*F*_n_). The COF is an empirical property of the contacting materials, in our case, of AFM tip and MoS_2 _nanotube, or MoS_2 _fullerene-like nano-onion, MoS_2 _single crystal or graphite. Accuracy of COF for a particular measurement was obtained from least-square fits. COF was determined in accordance with the known procedure [13].

#### Structure and surface morphology of MoS_2 _nanotubes

**Figure 1:**
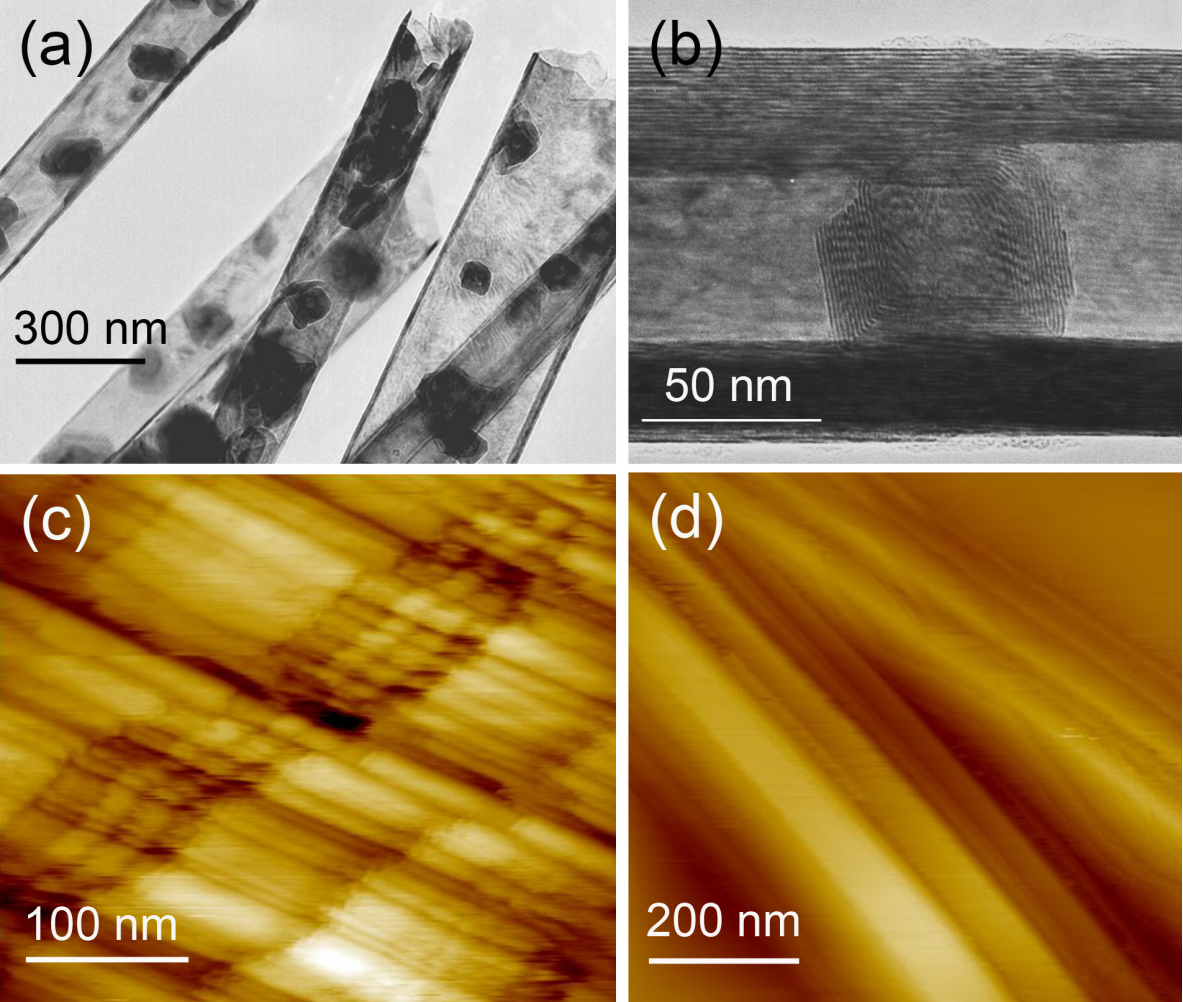
**The MoS_2 _m ama-tubes**. (**a**) Transmission electron micrograph of thin-walled nanotubes with encapsulated MoS_2 _nano-onions; (**b**) high-resolution image of the MoS_2 _nano-onion inside nanotube; (**c**) an STM image (*U*_T _= 0.5 V; *I*_T _= 0.5 nA) of a strongly undulated top layer with same cracks perpendicularly to the nanotube's axis; and (**d**) an STM image (*U*_T _= 0.5 V; *I*_T _= 0.5 nA) of two Mo_6_S_2_I_8 _nanowires, with exposed lamellar structure.

Recently reported MoS_2 _nanotubes, also named mama-tubes (Figure 1), represent a unique nanotube-hybrid nanomaterial with MoS_2 _spherical nanoparticles encapsulated in thin-walled MoS_2 _nanotubes [14]. The nanoparticles grow spontaneously in a confined geometry of nanotube reactors. The degree of the inner space occupied by nanoparticles differs from a sample to sample and depends on local conditions during the sulfurization process of the Mo_6_S_2_I_8_ starting material.

Encapsulated MoS_2 _fullerene-like nano-onions range from a few nanometers in diameter up to more than 100 nm (Figure 1a). Their shape can be quasi-spherical or partially facetted (Figure 1b). The STM reveals a modulation of topography at a nanometer scale (Figure 1c) explained as undulations of the (001) MoS_2 _basal planes with buried plane edges. This unique morphology appears due to a minimization of surface energy during the process of the nanotube formation, when quasi one-dimensional Mo_6_S_2_I_8 _needles (Figure 1d) are transformed to the curved two-dimensional MoS_2 _layers. Thickness of the walls is typically around 10 nm.

#### Friction measured on single MoS_2 _nanotubes laid flat on graphite (HOPG)

**Figure 2:**
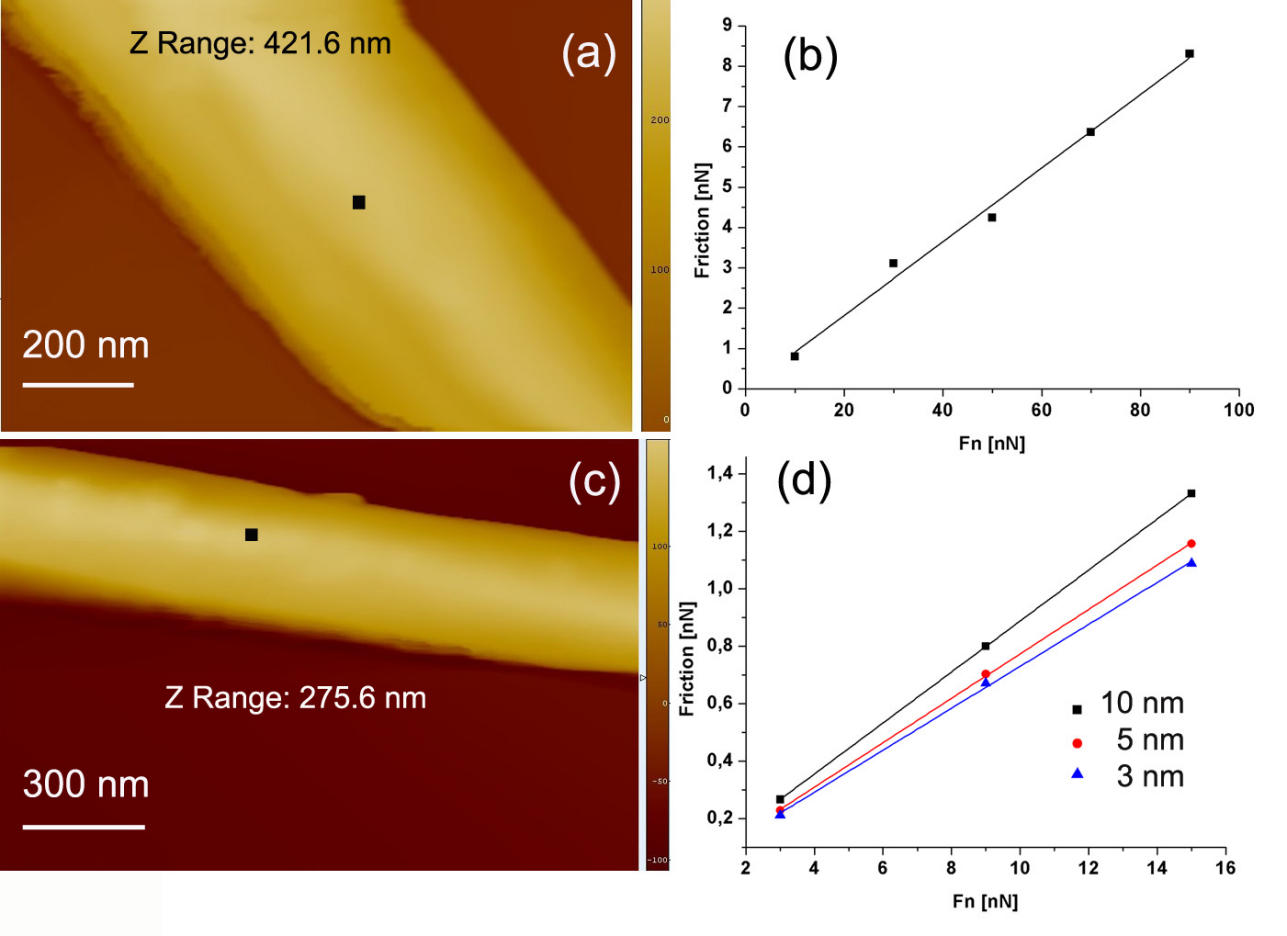
**AFM images of MoS_2 _nanotubes laid flat on the HOPG and friction results**. (**a**) The nanotube, 230 nm in diameter, with marked place of the friction test; (**b**) the corresponding friction results using loads from 15 to 90 nN, COF 0.091 ± 0.005; (**c**) the nanotube, 240 nm in diameter, where three scan areas were tested; and (**d**) the corresponding friction results using loads from 3 to 15 nN, average COF 0.080 ± 0.006.

The linear dependency of friction on load was tested on two nanotubes shown in Figure 2. The first nanotube, 230 nm in diameter (Figure 2a, b), was tested at relatively high loads (15, 30, 50, 70, and 90 nN). The scan area was 10 × 10 nm; the scan speed, 20 nm/s. The coefficient of friction determined from the slope of the linear fit, was 0.091 ± 0.005. Lower loads (3, 9, and 15 nN) were applied on the second tube, which is 240 nm in diameter. Three different scan areas using a scan speed of 20 nm/s were tested (Figure 2c, d). Corresponding values of COF were found to be slightly increased with the scan area: 0.073 ± 0.002 for (3 × 3 nm), 0.077 ± 0.001 for (5 × 5 nm), and 0.089 ± 0.001 for (10 × 10 nm). The changes can be attributed to the local effects of surface topography, where a larger scan area can bring more surface defects. Friction results at all applied loads show linear regime of friction. Lower loads were selected for further experiments.

#### Friction measured on single MoS_2 _nanotubes with weak interaction with the underlying substrate

**Figure 3:**
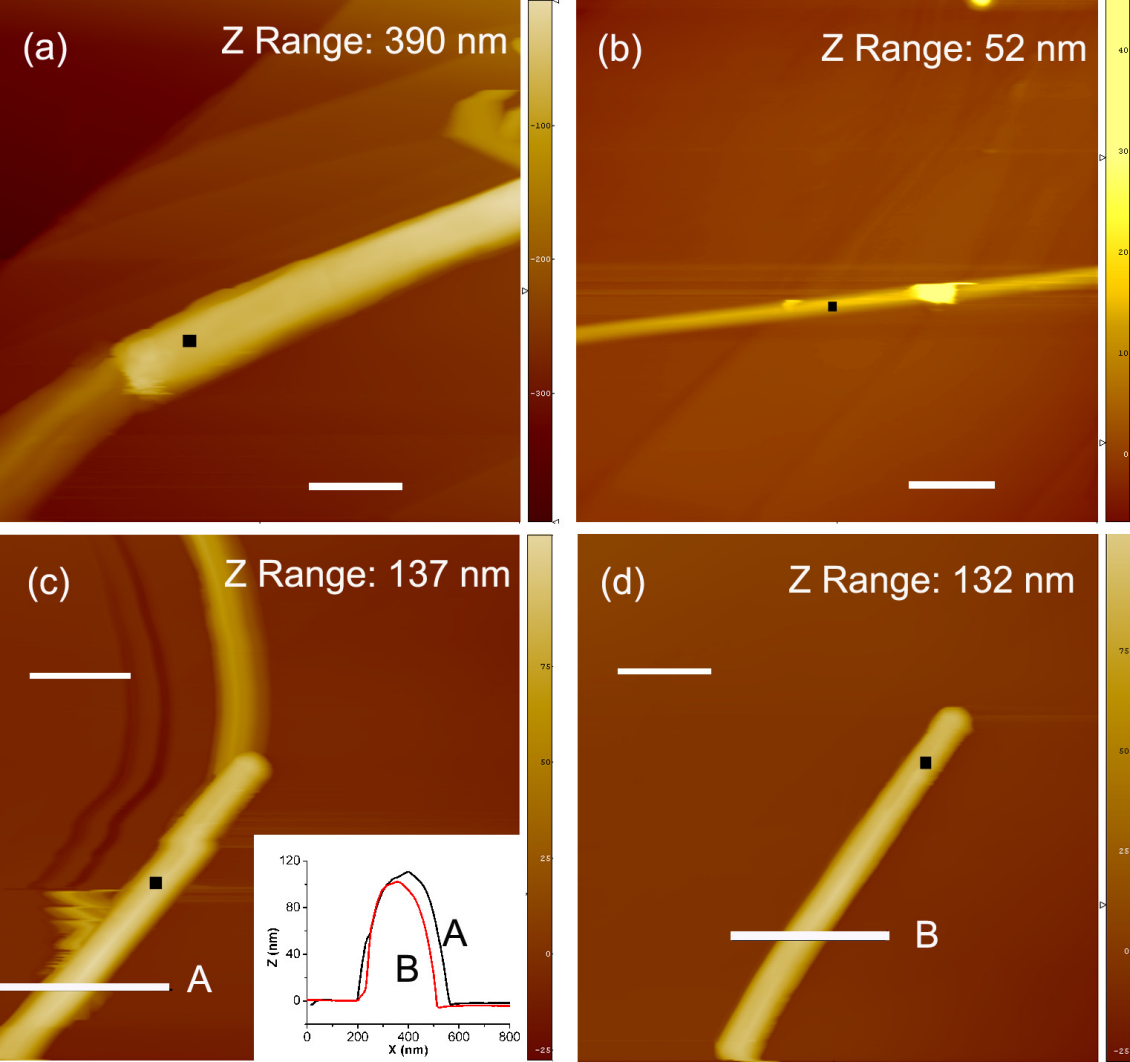
**AFM images of three MoS_2 _nanotubes**. There is low interaction strength between the nanotubes and underlying HOPG substrate and the corresponding values of COF with marked areas of the friction measurements; scale bars = 500 nm. (**a**) A kink of the tube, 200 nm in diameter (trace image), COF (0.018 ± 0.004); (**b**) the nanotube, 14 nm in diameter, laid over surface steps, COF (0.022 ± 0.005); (**c**) partially detached nanotube, 110 nm in diameter, with the line profile A (inset), COF (0.033 ± 0.001); and (**d**) the same nanotubes put into contact with substrate with the line profile B (inset), COF (0.057 ± 0.01).

Friction has been measured on nanotubes (Figure 3), such as tubes with kinks (Figure 3a), a tube laid over surface steps (Figure 3b), and partially detached tubes (Figure 3c). Several times, lower COFs were obtained; 0.018 ± 0.004 for the tube (Figure 3a), 0.0223 ± 30.005 for the tube (Figure 3b), and 0.033 ± 0.001 for the tube (Figure 3c), in comparison with well-supported nanotubes. This large decrease in friction with respect to values obtained on the tube laid flat on the substrate can be explained by the dragging of tubes by the AFM tip. The dragging is visible from a change of projection of the kink angle from the 20° in trace image (Figure 3a) to the 25° in the retrace image (not presented) and from horizontal shifts of the tube image in Figure 3c during the scanning process. Figure 3c, d shows the same nanotube before and after the first friction test. The interaction strength was changed, and the tube became well attached, presumably during the zoom out process when the position of the tube was controlled. The second friction test on the same nanotubes revealed a COF of 0.057 ± 0.01, which is doubled with respect to the first value. The corresponding line profiles (Figure 3c, inset) of the detached tube (profile A) and the well-attached one (profile B) show the difference in heights (10 nm), evidencing that in the first friction measurement, the tube was separated from the substrate for this value.

Although a trivial explanation, the interaction strength between the nanotube and underlying substrate, which changes the value of COF for several times, has a drastic influence on the use of nanomaterials as lubricants. The effect explains the still confusing phenomena [12] of higher friction typical of intracrystalline slip (0.06) than of intercrystalline slip (0.025) obtained for thin and flat MoS_2 _crystals. The values of COF show an also unexpected similarity, revealing a mechanism of friction, which is beyond the shape effects. On the first view, and neglecting role of defects, a shear (intracrystalline slip) should not differ from intercrystalline slip. It should be even less energetically costive due to known easy shear of basal planes of the MoS_2 _crystal structure parallel to the sliding direction leading to superlubricity [2], but just the opposite trend was reported [12]. Our results reveal that the interaction strength between the nanotube and underlying substrate plays a crucial role in the intercrystalline slip. Weak interaction prevents that the shear deformations would contribute to the energy cost in the friction process. The energy released during the friction process cannot dissipate to the substrate as easy as in intracrystalline slip, which is what results in lower consumption of energy by gliding and therefore to lower COF. Local electronic perturbation is also possible. Periodic crystal potential and electron dipole oscillations intensified by breaking of bonds between atoms of counterparts are at the origin of electron-phonon coupling; therefore, a local increase of temperature is expected.

Our experiments were performed in ultra-high vacuum, but the results can help to explain poorly understood instantaneous increase of friction in cases when MoS_2 _is exposed to water vapor. We propose the possible explanation that water condensates in nanovoids among MoS_2 _flakes even at low vapor pressure and in accordance with Kelvin equation [15], it remains there, where by surface tension, it bonds the MoS_2 _flakes together and influence the interaction strength between them. The intercrystalline slip becomes energetically more costly, and consequently, the friction increases.

#### Friction and angle of scanning

**Figure 4:**
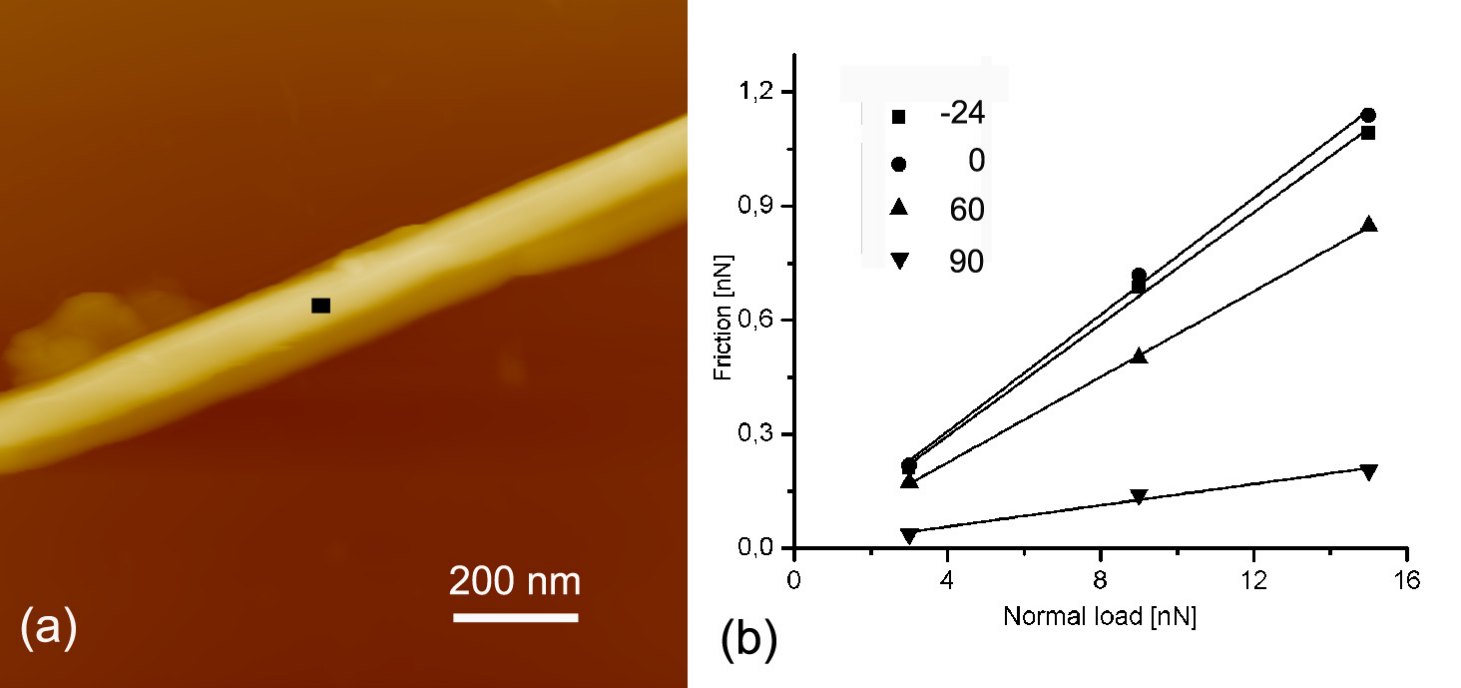
**The MoS_2 _nanotube 170 nm in diameter**. (**a**) Topography taken at 3 nN with marked area of friction testing and (**b**) friction results as a function of angle between the scan direction and the nanotube's axis.

One can easily predict that a high aspect ratio of MoS_2 _nanotubes and surface corrugation influence COF in a way that it depends on a direction of scanning with regard to a nanotube axis. This prediction was tested on the nanotube, 170 nm in diameter (Figure 4a), using a scan area (5 × 5 nm), a scan velocity of 12.5 nm/s, and loads of 3, 9, and 15 nN. The AFM tip scanned parallel to the tube axis (0°) and at three relative angles, -24°, 60°, and 90°. While no clear difference in COF was found for the scans at 0° and -24° angles, where COF was 0.077 ± 0.004 and 0.074 ± 0.003, respectively, a strong decrease of COF was found at larger relative angles, i.e., 0.056 ± 0.001 at 60°, and 0.014 ± 0.002 at 90°. It is important to note that the scan area was much smaller than the tube diameter; therefore, the curvature of the tube could not influence the friction. One possible reason for the decrease of COF with the angle is a spontaneous surface texturing of MoS_2 _nanotubes (Figure 1c) with protruding shape features parallel to the tube axis. Some hidden local peculiarities of the scan area could also be the reason; as an example, some variations in probability for intracrystalline slip due to structural defects. No wear effects were detected in topography images. Very low COF obtained at 90° is close to the ultra-low friction reported for MoS_2_-MoS_2 _interface [2].

#### Deformations of the MoS_2 _nanotube wall

**Figure 5:**
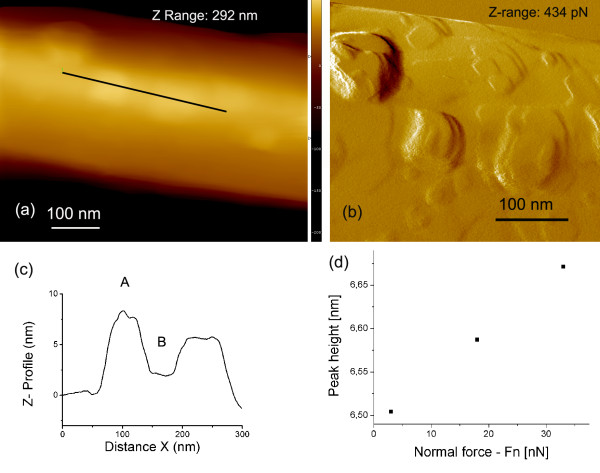
**MoS_2 _mama-tube well supported by the HOPG**. (**a**) AFM image in contact mode with brighter areas indicating position of MoS_2 _nano-onions below the thin nanotube wall. Position of line profile is marked. (**b**) The corresponding magnified normal-force image with clearly resolved nanoparticles below the surface layer. (**c**) A typical line profile with marked positions of the measured peak (A) and the valley (B) between two underlying particles and (**d**) bending deformation as a function of load.

Wall thickness of MoS_2 _nanotubes with imbedded MoS_2 _fullerene-like nano-onions is typically in a range around 10 nm [16]. Our assumption that the interaction strength between a nano-object and underlying substrate influences the COF was tested and proved on a tube 330 nm in diameter (Figure 5a). We focused on a specific place between two protrusions visible in normal force image (Figure 5b), which indicate position of imbedded MoS_2 _nano-onions bellow the nanotube wall. This particular area, marked with (B) in Figure 5c, resembles the situation of a nanotube with a weak interaction strength by its substrate, although only a part of the upper wall is without a direct support. Two scan areas (5 × 5 nm and 10 × 10 nm) were measured. The average value of COF over four measurements was 0.04 ± 0.002. The same kind of the test was performed at the areas above imbedded nano-onions, where the average value of COF over six measurements was 0.07 ± 0.01.

We have also tested bending of the nanotube wall under the AFM tip as a function of the position of imbedded nano-onions. The wall is so thin that one can expect that a deformation of the wall under AFM load is larger in areas without 'support' of nano-onions. The line profile (Figure 5a) over two nanoparticles indeed reveals a 'camel' shape (Figure 5c). Measuring the distance between the top of the peak (A) and the valley between two peaks (B) at different loads in a range between 3 and 33 nN shows that the distance (i.e., the peak height or the valley depth) changes in a linear way with the load (Figure 5d) revealing the elastic deformation.

#### Friction measured on a single MoS_2 _fullerene-like nano-onion

**Figure 6:**
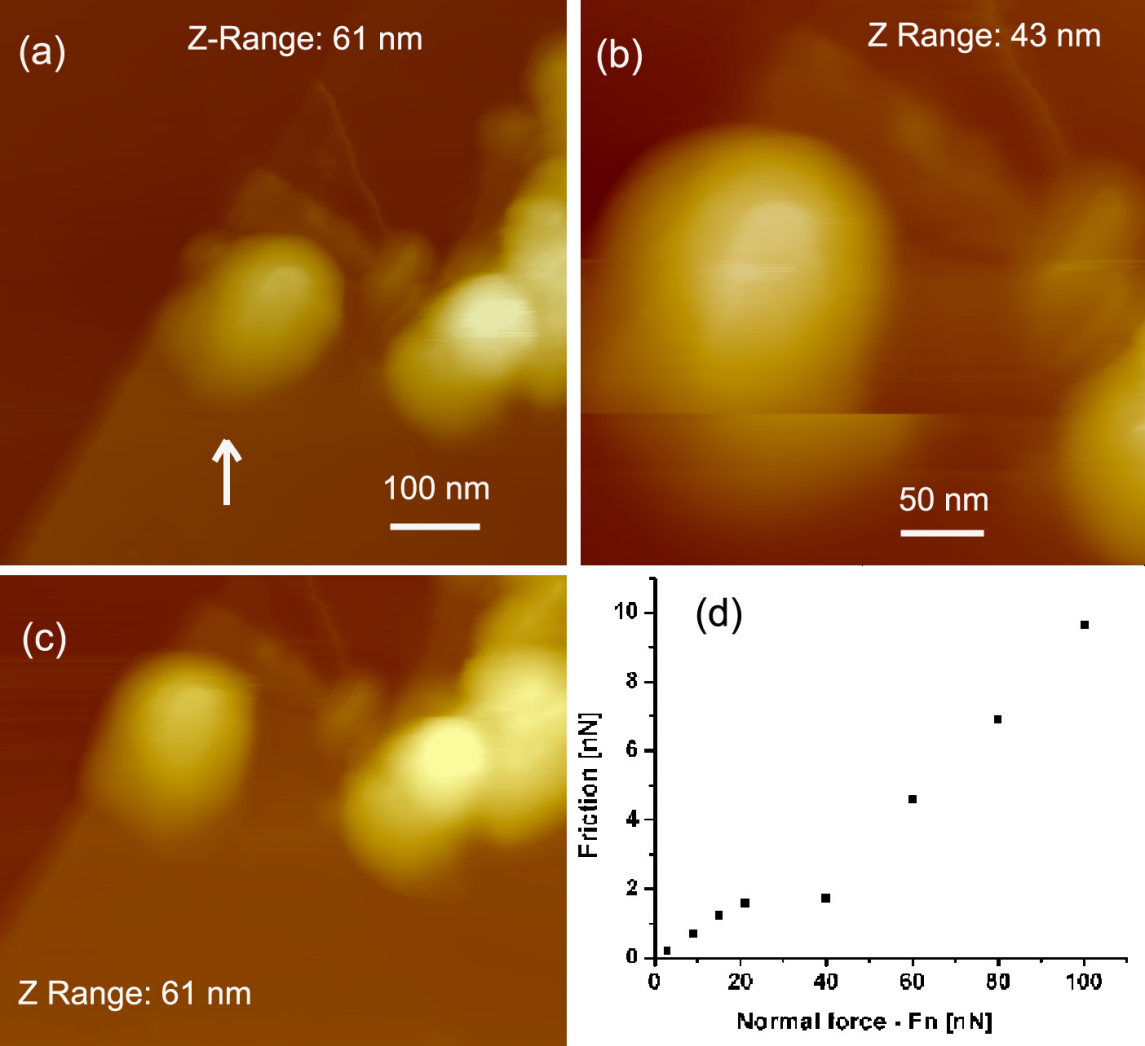
**MoS_2 _nano-onions**. (**a**) A group of MoS_2 _nano-onions before friction test. (**b**) The selected nano-onion (marked with arrow in the previous image), 30 nm in diameter, shifted for 35 nm toward the left during the second scanning. (**c**) The same group of nano-onions after the friction test with shifted position of the selected nano-onion for 50 nm toward the left and rotation for 10° around the axis perpendicular to the substrate. (**d**) Friction as a function of load with two slopes revealing COF of 0.077 ± 0.004 and 0.12 ± 0.003, respectively.

Friction was measured also on the single MoS_2 _nano-onion released from mama-tubes. A group of nano-onions on HOPG is shown in Figure 6. The diameter of the selected (arrow) nano-onion (Figure 6a) was 30 nm. We observed that the nano-onion drifted toward the left for 35 nm from the first scan taken at 3 nN to the second one (Figure 6b). AFM tip was then put in contact with the new position of the center of the same nano-onion and friction testing started using the scan area of 10 × 10 nm and the scan velocity of 20 nm/s. Friction as a function of load, which was increased from 3 to 100 nN (Figure 6c), clearly shows a change of the slope at the 40-nN load. The transition from the slope revealing COF of 0.077 ± 0.004, typical for MoS_2 _from previous measurements, to the slope for COF of 0.12 ± 0.003 can be explained by the drift of the nano-onion with respect to the AFM tip. The tip slipped from the nano-onion to the HOPG substrate. This explanation is supported with the AFM image of the same group of nano-onions after the friction experiment, which reveals a further drift of the tested nano-onion toward the left for 15 nm, enough that the AFM tip had fallen down to the substrate. Consequently, the position of the laser was changed due to a large disturbance in height of the AFM tip. Such a disturbance explains why extrapolation of the measured results toward zero friction has gotten inappropriate in this range of the spectrum. The results show a linear dependence of friction on load confirming the Coulomb law. A comparison of Figure 6a, c reveals that only the nano-onion, which was the object of the friction testing, was moved. Resolution of the AFM images was sufficient that both gliding and rolling processes were evidenced.

## Discussion

Based on values of the COF, the nanotubes can be divided to those with a strong interaction between the nanotube and the underlying substrate, and to 'semi' self-standing ones. The term semi is used because the measured area was located at the site where the tube was detached from the substrate, although other parts of the same nanotube were well-supported by the substrate. The average COF value of the supported nanotubes is found to be 0.08 ± 0.02, while COF of semi self-standing tubes is a few times lower, i.e., 0.02 ± 0.005. It is known that friction is related to the bonding energy between the surfaces, mainly to the adsorption energy of the sliding surfaces on each other [17]. Moreover, the atomic layers below the gliding surfaces contribute to the dissipation process significantly [18]. Our finding that the substrate, which is for the nanotubes' diameter apart from the studied contact change friction between the nanotubes and the AFM tip, is in line with the model, which considers plastic deformation of the contact as a prime actor in dissipation. In semi self-standing tubes, such dissipation of energy in limited, intracrystalline shear stress is minimized and the tubes behave rigidly. Lower energy is released in the friction process, what together with dragging process explains very low COF.

It is important to note that although the higher COF is found on well-supported nanotubes, this value (0.08 ± 0.02) is still much below the average COF obtained at the same testing conditions on air-cleaved MoS_2 _single crystal (0.115 ± 0.003) and on HOPG (0.16 ± 0.01). Results found on single crystals are in the range of published friction data for vacuum conditions (different testing methods), 0.15 to 0.3 for MoS_2 _[19] and 0.06 to 0.3 for graphite [20], and are higher than the friction coefficient on freshly cleaved MoS_2 _single crystal (0.023 ± 0.001) measured with Si3N4 AFM tip [13]. It is interesting to note that the last value perfectly matches our results obtained on weakly supported nanotubes.

Friction measurements on a single MoS_2 _fullerene-like nano-onion revealed in a range of accuracy the same COF (0.077 ± 0.004) as found on well-supported MoS_2 _nanotubes. This indicates that the shape difference between cylindrical and spherical geometry does not play the dominant role in friction mechanism of MoS_2_.

## Conclusions

For the first time, friction was measured on a single MoS_2 _nanotube and on a single MoS_2 _nano-onion, both on HOPG substrate. Experiments were performed in UHV at room temperature. We found that the coefficients of friction between silicon AFM tip and MoS_2 _nanotube or MoS_2 _nano-onion are much below the relevant values for flat single crystal MoS_2 _or graphite. Further, we found indications that friction at the nanoscale depends strongly on interaction strength between the nanotube and underlying substrate, which is what is explained with shear deformation and dissipation of energy. The MoS_2 _nanotubes with high interaction strength revealed up to four times larger COF (0.08 ± 0.02) than weakly supported tubes with COF of 0.023 ± 0.005. The results explain the contradictory old phenomena of higher friction typical for intracrystalline slip than for intercrystalline slip. They contribute to understanding of typically, highly scattered results using nanomaterials as lubricants. We evidenced that a rolling mechanism of MoS_2 _fullerene-like nano-onions is possible at low loads.

## Competing interests

The authors declare that they have no competing interests.

## Authors' contributions

JJ conducted measurements and contributed in the interpretation of results. MR supervised the overall study and wrote the manuscript. All authors read and approved the final manuscript.

## Authors' information

JJ, BSc is connected with the Solid State Physics Department, Jozef Stefan Institute and Centre of Excellence NAMASTE. MR is a senior researcher at the Solid State Physics Department, Jozef Stefan Institute and Centre of Excellence NAMASTE.
